# Posterior tibial slope (PTS) ≥ 10 degrees is a risk factor for further anterior cruciate ligament (ACL) injury; BMI is not

**DOI:** 10.1007/s00590-022-03406-9

**Published:** 2022-10-06

**Authors:** Ali Fares, Clément Horteur, Morad Abou Al Ezz, Alexandre Hardy, Brice Rubens-Duval, Karam Karam, Benoit Gaulin, Regis Pailhe

**Affiliations:** 1 Department of Osteoarthritis and Sport Surgery, Grenoble-Alpes CHU, South Teaching Hospital, Kimberley Avenue, BP 338, 38434 Échirolles Cedex, France; 2 Chirurgie du Sport, Clinique du Sport Paris V, Ramsay-Générale de Santé, 75005 Paris, France

**Keywords:** Further ACL rupture, Posterior tibial slope, Knee radiograph, Body mass index

## Abstract

**Purpose:**

This case–control study aimed to assess the influence of BMI and PTS on subsequent ACL injury affecting either ACL graft or the native ACL of the contralateral knee after primary ACL reconstruction.

**Methods:**

A retrospective case–control study was performed using a cohort of patients who underwent arthroscopic ACL reconstruction between 2010 and 2020 using the same surgical procedure: Hamstring tendon autograft. The study group (group I) included all the patients (*n* = 94) during this period who sustained a subsequent ACL injury. The control group (group II) consisted of 94 patients randomly selected (matched Group I in terms of sex, age, and ACL graft) who did not sustain any further ACL injury. PTS was measured by two blinded surgeons on lateral knee view radiographs of the operated knee after primary ACL. BMI in kg/m^2^ was measured during the preoperative anesthesia consultation. Exclusion criteria were: non-true or rotated lateral knee radiographs of the operated knee post-ACLR, associated knee ligament injury requiring surgical management, iterative knee surgeries, open growth plate, and related fracture.

**Results:**

The mean posterior tibial slope in group I was 7.5° ± 2.9, and 7.2° ± 2.0 in group II. A PTS angle cutoff was set at 10 degrees. The rate of patients showing a PTS ≥ 10° was significantly higher in group I compared to group II (*p* < 0.01). Patients with PTS ≥ 10° were 5.7 times more likely to sustain a subsequent ACL injury, (OR: 5.7 95% CI[1.858–17.486]). The Average BMI in group I was 24.5 ± 3.7 kg.m^−2^ compared to group II which was 23.3 ± 3.0 kg.m^−2^. There were no significant differences in any of the four BMI categories between both groups (*p* value 0.289). A series of BMI cut-offs were also analyzed at 23 to30 kg/m^2^, and there was no significant difference between both groups.

**Conclusions:**

A posterior tibial slope equal to or above 10 degrees measured on lateral knee radiographs was associated with 5.7 times higher risk of ACL graft rupture or contralateral native ACL injury; however, BMI was not.

## Introduction

An anterior cruciate ligament (ACL) injury and graft failure are multifactorial events influenced by modifiable and non-modifiable risk factors [1]. The posterior tibial slope (PTS) is one of the important non-modifiable anatomical factors that influence knee biomechanics [2] unless corrected surgically. Its variations affect the kinematics and stability of the knee in such a way that an increased PTS is now considered a risk factor for primary ACL injury [3, 4]. Another important factor, the body mass index (BMI), is a significant modifiable risk factor that has a well-known impact on primary ACL injury [5].

However, there are different points of view in the literature concerning the contribution of the BMI as well as PTS [6–11] in iterative ACL injury. Some investigators [12, 13] find no correlation between the rate of ACL graft rapture and the PTS values. This is in contrast with the findings that further ACL injury may be affected by the medial tibial slope, the lateral tibial slope, or both [8–11, 14–17].

Reinjury rates are a primary concern in any surgical technique or rehabilitation protocol, and they remain a great challenge for surgeons in the setting of multiple revisions [18].

Considering the risk of iterative (7%) or contralateral (8%) ACL rupture as reported in a recent systematic review [19], it is essential to investigate its risk factors and draw a clear pathway for surgical indications and patients’ rehabilitation. This retrospective case–control study aimed to assess the influence of the BMI and PTS on subsequent ACL injury, including the ACL graft or the native ACL of the contralateral knee after primary ACL reconstruction. We hypothesized that a new PTS cutoff at 10 degrees would be a risk factor for further ACL rupture. We also investigate the role of the BMI in this group of patients to draw potential conclusions.

## Methods and materials

This is a monocentric observational retrospective case–control study undertaken according to the principles of the Helsinki declaration. All patients included gave informed consent. The Regional Ethics Committee reviewed (IRB00010835) and approved the study protocol.

### Study design and samples

All patients who had primary ACL reconstruction at our university institution between 2010 and 2020 were screened for eligibility for this retrospective case–control study using the Crystal-Link® software.

Consequently, 2712 patients post-ACLR were screened. Among them, 108 patients had been treated with a classic double-bundle hamstring graft and sustained further ACL injury (ipsilateral graft rupture or contralateral ACL tear) within the study period. Of those, patients excluded had non-true or rotated lateral knee radiographs of the operated knee post-ACLR, associated knee ligament injury requiring surgical management, iterative knee surgeries, open growth plate, and related fracture. Consequently, 14 patients were excluded and 94 patients remained to form the study group (Group I). All subsequent ACL injuries were confirmed by clinical examination (Lachman and pivot-shift test), instrumented laxity tests with the KT-1000 arthrometer (Medmetric Corp ®, San Diego, California) on both knees, and magnetic resonance imaging (MRI) of the affected knee.

**Fig. 1:**
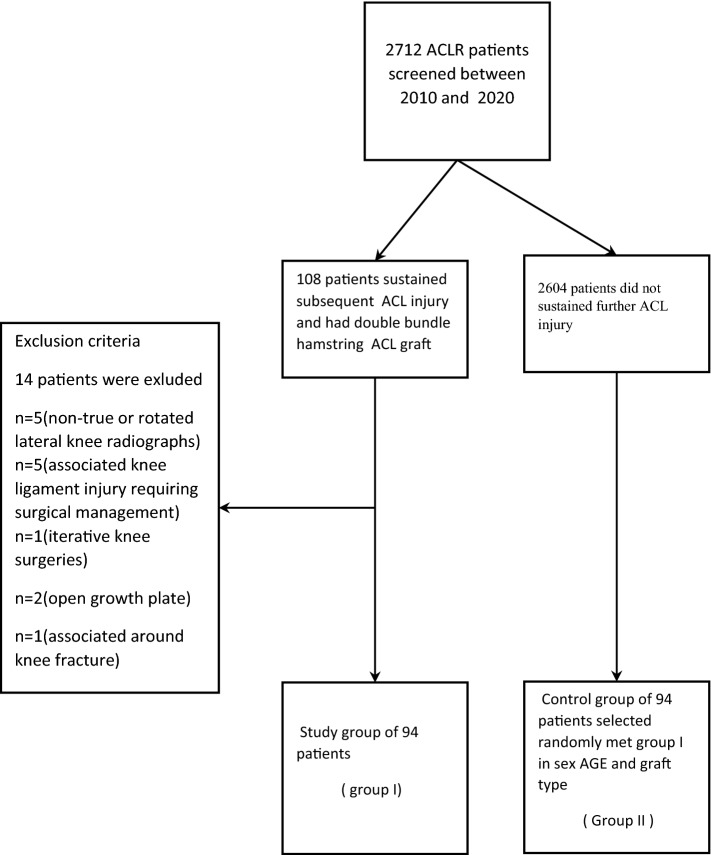
A flow chart showing the distribution of groups. (ACL: anterior cruciate ligament; CACL: contralateral anterior cruciate ligament)

A control group of 94 patients selected randomly from the same cohort matched group I in age, sex, and graft type. These patients were followed up according to their medical charts and a phone call by one of the authors while performing the study to confirm no further injury for a minimum of two years(mean 6.4 + − 2.2) and no subsequent ACL injury was diagnosed (Fig. 1, flowchart).

### Procedures

**Fig. 2:**
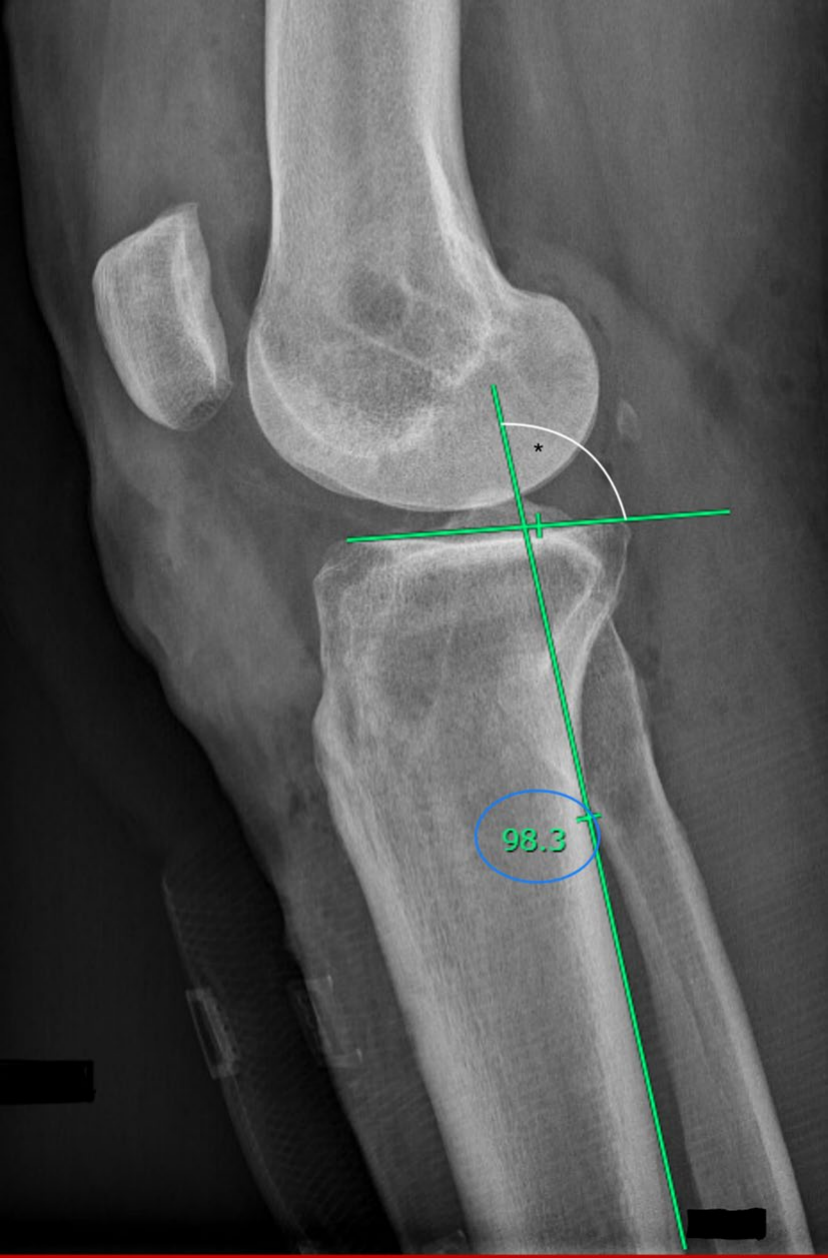
Radiograph showing the method of measuring the PTS. The circle identifies the value of the angle (*) detected between the tibial axis line and the tangent line drawn over the tibial plateau, PTS° = *°− 90° = 98.3°–90° = 8,3°

All lateral knee radiographs of the operated knee were verified for eligibility and then sent to two blinded senior orthopedic surgeons to measure the posterior tibial slope, using Picture Archiving and Communication System (PACS®) software (Fig. 2). PTS is defined as the angle between the tibial anatomic axis and a tangent line drawn over the tibial plateau minus 90 ͦ. The anatomic axis of the tibia was determined using the posterior tibial cortex method described by Hohmann et al. [2] as shown in Fig. 2 and was measured after the index procedure, on the operated knee post-operative radiograph. Good inter-and intra-observer reliability in the measurements was obtained.

Globally the mean posterior tibial slope is 5.3 to 6.3 [20]. No consensus was found in the literature on an upper threshold of tibial slope to minimize the risk of recurrent [21] ACL injury. A PTS angle cutoff was set at 10 degrees to compare groups according to high values of PTS. To our knowledge, this is the first study in which a cutoff is set at 10 degrees.

BMI was calculated as values in kg/m^2^ and was measured during the anesthesia preoperative consultation at our University Hospital.

### Surgical procedure and rehabilitation

All procedures were arthroscopically assisted and performed under loco-regional or general anesthesia; the patient was positioned in dorsal decubitus with a pneumatic thigh tourniquet. The intra-articular procedure was strictly identical in both groups. Tibial and femoral tunnels were defined by positioning a wire using an outside-in method with an angulated guide. Tunnels were then drilled using cannulated reamers of diameters corresponding to the graft width followed by fixation of the double-stranded autograft using semitendinosus and gracilis tendons. All patients followed the same standard rehabilitation protocol. Follow-up visits were proposed for 45 days, 3 months, 6 months, 1 year, and 2 years after the surgery. The rehabilitation protocol was performed by the national guidelines from 2008. It was divided into 5 periods running from the surgery day until the return to sports practice without any restriction 8 to 9 months later [22]. In the case of meniscal suture, knee flexion while weight-bearing was limited to 120° for two months postoperatively.

### Statistical analysis plan

Statistical analysis was performed using IBM SPSS version 25®.

Descriptive analysis of qualitative variables was expressed in percentages in each category. Quantitative variables were summarized in tables using descriptive statistics (analyzed number n, mean, standard deviation, minimum, maximum).

Bivariate analysis was conducted to test the statistical difference between Group I and Group II. Tests used in bivariate settings were the Pearson correlation test, Chi-square test, Fisher exact test, and ANOVA test. All statistical tests were two-sided, and the significance level was set at 5%.

## Results

### Population characteristics are summarized in (Table 1).

**Table 1:** Demographic characteristics

		Group I Further ACL injury (*n* = 94)	Group II Control group No further ACL injury (*n* = 94)	*p* value
Age at first anterior cruciate ligament injury (year)	Number of subjects	94	94	0.617*
	Mean ± SD	24.5 ± 8.3	25.1 ± 7.5	
	Min–Max	13.5–48.7	14.2–46.8	
Gender	Male	71	67	0.621†
		75.5%	71.3%	
	Female	23	27	
		24.5%	28.7%	
No further injury Follow-up (year)	Number of subjects	NA	94	NA
	Mean ± SD		6.4 ± 2.2	
	Min–Max		2–11	
Time to further anterior cruciate ligament injury (month)	Number of subjects	94	NA	NA
	Mean ± SD	30.7 ± 23.6		
	Min–Max	3–101		

*p value was calculated using one-way ANOVA

^†^*p* value was calculated using Chi-square test or Fisher exact test

*ACL* anterior cruciate ligament

Both groups were comparable in terms of age (24.5 vs. 25.1 in group I and group II, respectively, *p* value 0.617) and sex (75.5% men 24.5% women vs. 71.3% men 28.7% women in groups I and II, respectively, *p* value 0.621). The mean time for subsequent ACL injury in group I was 30.7 + − 22.6 months, and the mean follow-up duration for group II was 6.4 years +  − 2.2.

### Posterior tibial slope (Table 2)

**Table 2:** Analysis of the posterior tibial slope

		Group I Further ACL injury (*n* = 94)	Group II Control group No further ACL injury (*n* = 94)	*p* value
Posterior tibial slope (PTS)	Number of subjects	94	94	0.412*
	Mean ± SD	7.5 ± 2.9	7.2 ± 2.0	
	Min–Max	1.5 -14.2	2.2–11.8	
Posterior tibial slope (PTS)	< 10°	75	90	0.001†
		79.8%	95.7%	
	≥ 10°	19	4	
		20.2%	4.3%	
Odds ratio		5.7 95% CI (1.858–17.486)		

*p value was calculated using one-way ANOVA

^†^*p* value was calculated using Chi-square test or Fisher exact test

*ACL* anterior cruciate ligament

The mean posterior tibial slope in group I was 7.5° ± 2.9, and 7.2° ± 2.0 in group II. There was no significant difference between both PTS means (*p* value = 0.412). The rate of patients showing a PTS ≥ 10° was significantly higher in group I compared to group II (20,2 vs. 4,3%, respectively, *p* < 0.01).

The patients with increased PTS > 10° were found to be 5.7 times more likely to sustain a subsequent ACL injury, either ACL graft rupture or contralateral ACL rupture (OR: 5.7 95% CI[1.858–17.486]).

### Body mass index (Table 3)

**Table 3:** Analysis of the body mass index categories

		Group I Further ACL injury (*n* = 94)	Group II Control group No further ACL injury (*n* = 94)	*p* value
Body mass index categories	Underweight (below 18.5)	1	2	0.289†
		1.1%	2.1%	
	Normal (between 18.5 and 24.9)	57	66	
		60.6%	70.2%	
	Pre-obesity (25.0–29.9)	28	23	
		29.8%	24.5%	
	Obesity class I (30.0—34.9)	8	3	
		8.5%	3.2%	
Body mass index (Kg/m^2^)	Number of subjects	94	94	0.012*
	Mean ± SD	24.5 ± 3.7	23.3 ± 3.0	
	Min–Max	17.0–39.2	17.5–32.4	

*p value was calculated using one-way ANOVA

^†^*p* value was calculated using Chi-square test or Fisher exact test

Average BMI was higher in group I (24.5 ± 3.7 kg.m^−2^) compared to group II (23.3 ± 3.0 kg.m^−2^). (*p* = 0.012). BMI values were classified into four ranges < 18.5, 18.5–24.9, 25–29.9, and > 30 in both groups. However, there were no significant differences in any of the four categories between both groups (p value 0.289). A series of tests according to BMI cutoff was made at 23, 24, 25, 26, 27, 28, 29, and 30 kg/m^2^. There was no difference between both groups in terms of all cutoff values.

### BMI PTS correlation (Table 4, and 5)

**Table 4:** Relation of posterior tibial slope to age, gender, body mass index and time to further ACL injury among patients in Group I

			Posterior Tibial Slope (PTS)	*p* value
Age		Number of subjects	94	0.584^¥^
		Pearson correlation	− 0.057	
Gender	Male	Number of subjects	71	0.312*
		Mean ± SD	7.7 ± 2.9	
	Female	Number of subjects	23	
		Mean ± SD	7.0 ± 2.8	
Body mass index (Kg/m^2^)		Number of subjects	94	0.622^¥^
		Pearson correlation	− 0.051	
Time to further anterior cruciate ligament injury		Number of subjects	94	0.426^¥^
		Pearson correlation	0.083	

Among patients who performed ACL rupture, PTS was not associated with age (*p* = 0.584), gender (0.312), BMI (*p* = 0.622), and time to further anterior cruciate ligament injury (*p* = 0.426) (Table 5)

*p value was calculated using independent t test

^¥^*p* value was calculated using Pearson correlation

**Table 5:** Relation of posterior tibial slope to age, gender, and body mass index among patients in Control Group II

			Posterior tibial slope (PTS)	*p* value
Age		Number of subjects	94	0.684^¥^
		Pearson correlation	0.043	
Gender	Male	Number of subjects	63	0.729
		Mean ± SD	7.2 ± 2.1	
	Female	Number of subjects	26	
		Mean ± SD	7.1 ± 1.8	
Body mass index (Kg/m^2^)		Number of subjects	94	0.901^¥^
		Pearson correlation	0.013	

*p value was calculated using independent *t*-test

¥*p* value was calculated using Pearson correlation

Among the patients in group I, there was no correlation found between PTS and age (*p* = 0.584), gender (0.312), BMI (*p* = 0.622), or time to subsequent anterior cruciate ligament injury (*p* = 0.426). Similarly, among the patients in group II, PTS was not correlated with age (*p* = 0.684), gender (0.729), or BMI (*p* = 0.901).

## Discussion

The most important finding of this study was that for subsequent ACL injury, BMI is not a risk factor. However, a PTS ≥ 10 degrees is a risk factor affecting either the ACL graft of the operated knee or the native ligament of the contralateral knee.

Among the 188 patients, there was no significant difference between both groups, neither in terms of the 4 BMI ranges nor in terms of BMI cut-offs from 23 to 30 kg/m^2^. Therefore, BMI was not found to be a continuous risk factor for subsequent ACL injury. Similar results were found in the studies of Funabashi et al. [23] and Eivind et al. [24] in which BMI had no impact on recurrent ACL injury. This may be due to the fact that our patient population included a majority of athletic patients with normal-range BMI. Another possible explanation is the fact that patients with a higher BMI will be less likely active, and therefore less prone to injury. In a Swedish and Norwegian population, Thorkell et al. [7] considered a BMI over 25 kg/m^2^ as a risk factor for early revision. In contrast, Person et al. [25] found that patients with BMI superior to 25 kg/m^2^ had a lower risk of ACL graft rupture compared with patients below 25 kg/m^2^. Surprisingly, Maletis et al. [26] reported on a series in which patients with BMI ranging from 30 to 35 kg/m^2^ and above 35 kg/m^2^ had a lower risk of recurrent ACL injury compared to patients with BMI under 30 kg/m^2^. In a recent systematic review, lower BMI was found to be a high risk for revision ACL surgery [6] also another systematic review found that in patients with BMI > 25 kg/m, the risk for revision surgery or contralateral ACL tear was lower [27]. Thus, in the case of iterative ACL injury, other risk factors must be identified. Nevertheless, surgeons should still recommend weight loss in this obese population because of the increased risk of developing knee osteoarthritis as BMI increases [28, 29].

The mean posterior tibial slope in both groups was approximately 7 degrees. This corroborates with results from the current literature describing the mean posterior tibial slope [20, 30]. Since there is no consensus regarding the superiority of PTS measurements on lateral radiographs or magnetic radiographic imaging [31], we preferred to make our measurements on lateral radiographs using the posterior cortex method [2]. This method has shown excellent reliability for both revision ACL and primary ACL patients and was later found to be the most reliable method to measure PTS [32].

The percentage of patients with excessive PTS ≥ 10° was higher in group 1 compared with those in group II (20% vs. 5%, respectively; *p* = 0.001; OR = 5.7). We can conclude from this that the patients with a markedly increased PTS ≥ 10° are associated with a 5.7 times higher risk of subsequent ACL injury. This is due to the increased anterior tibial translation that exerts a higher strain on the ACL or ACL graft [21, 33]. Many publications found that a PTS > 12° is associated with a higher risk of revision [21, 34–37]. This is described as the "12 degrees rule," negatively affecting the survival of ACL reconstruction [11, 35, 38]. A cohort of 330 subjects [39] found that patients who had a third ACL injury following previous revision ACL reconstruction had greater mean radiographic posterior tibial slope values than those who did not sustain a further ACL injury. Furthermore, Salmon et al. [35] illustrated a population of adolescents with a PTS > 12 degrees who were 11 times more likely to sustain an ACL graft failure. However, there is still no consensus on a threshold of tibial slope to minimize the risk of recurrent injury. Based on the average PTS in the normal population, which is usually inferior to 10 degrees, regardless of the method of measurement [21, 25], we decided to set a cutoff of 10 degrees. Ours was the first study to set this value as a cutoff.

Cooper et al. [40] reported that PTS is not a risk factor for further ACL injury. Similar findings by Hudek et al. [41] described no relationship between lateral or medial PTS measurements with MR images and recurrent ACL injuries. In addition, a prospective cohort study by Beynnon et al. [8] concluded that medial PTS was not associated with the risk of ACL injury. These studies were completed using MR measurements of the PTS. We opted to perform our measurements using post-operative lateral knee X-rays.

Our results showed that BMI did not affect the relationship between PTS and further ACL injury risk, as Pearson correlation was PTS, in contrast to other studies such as that of Katherine et al. [42] who found that an increase in BMI may increase the risk of ACL injury in the presence of increased lateral posterior tibial slope.

Because of the significant relationships found in our study, we recommend systematic measurement of the PTS in patients undergoing primary ACL reconstruction. For patients with high PTS, which was ≥ 10° in our study, patient counseling should be undertaken to convey the potentially increased risk of further ACL injury in either knee. An initial course of conservative management consisting of a structured rehabilitation program of proprioception and muscular strengthening may be a way of minimizing the increased risk. Discussion with patients about invasive procedures such as high tibial osteotomy HTO in cases of ACL graft rupture associated with a high PTS should be considered [11, 43, 44]. As a matter of fact, a cadaveric study by Imhoff et al. [45] suggests that knee kinematics can be improved in ACL-reconstructed knees, as well as in ACL-deficient knees, provided an ACL reconstruction is performed concomitantly. Similarly, Sonnery-Cottet et al. [44] reported that a combined ACL re-revision associated with a proximal tibial closing wedge osteotomy restores knee stability and function, following recurrent ACL ruptures associated with an increased PTS.

### Limitations

This study has several limitations: First, it is a retrospective monocentric study. Also, patients were operated on by several senior surgeons, but they used the same technique. Moreover, the number of patients with an elevated BMI is limited because most of the patients in the study population are athletic. Our two patient groups did not include information on meniscal injury management, including meniscectomy and repair. This is important because our institution applies a rehabilitation protocol for such patients. Furthermore, generalized joint laxity analyses, such as the Beighton score, were not informed in our patient populations. However, we correctly matched the patients for gender and age, and the follow-up period in the control group was 6.4 ± 2.2 years after primary ACL reconstruction. This corresponds to a duration that is three times longer than the duration to sustain another ACL injury in the study group (30.7 ± 23.6 months). A power analysis was not necessary considering it was a retrospective observational study.

## Future directions

Further studies to improve the relevance of these findings would be to design a prospective multicentric randomized study. More detailed demographic information may allow us to identify a subset of patients at a higher risk of further ACL rupture, such as profession, activity level, laxity scores, and concomitant knee injuries and prior operations, such as meniscal or cartilage involvement.

## Conclusion

A posterior tibial slope equal to or above 10 degrees measured on lateral knee radiographs was associated with a 5.7 times higher risk of ACL graft rupture or contralateral native ACL injury in patients who had hamstring autograft ACL reconstruction surgery; however, BMI was not noted as a proportional or exponential risk factor. Considering the increasing body of evidence supporting the role of PTS in ACL reconstruction outcomes, attention should be given to the identification of patients at risk of further ACL injury. Those patients should be advised about the potential benefits of additional precautions or surgical slope correction.

## Abbreviations

ACL: Anterior cruciate ligament
PTS: Posterior tibial slope
BMI: Body mass index
ACLR: Anterior cruciate ligament reconstruction
CACL: Contralateral anterior cruciate ligament

## Declarations

### Human or Animals rights

Humans were involved, and all procedures performed in studies involving human participants were in accordance with the ethical standards of the institutional and/or national research committee and with the 1964 Helsinki Declaration and its later amendments or comparable ethical standards. Informed consent was obtained from all individual participants included in the study. The Regional Ethics Committee (IRB00010835) reviewed and approved the study protocol on 21/04/2022, see the certificate in the supplemental materials.
